# KPC-3–Producing *Serratia marcescens* Outbreak between Acute and Long-Term Care Facilities, Florida, USA

**DOI:** 10.3201/eid2611.202203

**Published:** 2020-11

**Authors:** Adriana Jimenez, Lilian M. Abbo, Octavio Martinez, Bhavarth Shukla, Kathleen Sposato, Alina Iovleva, Erin Louise Fowler, Christi Lee McElheny, Yohei Doi

**Affiliations:** Jackson Health System, Miami, Florida, USA (A. Jimenez, L. M. Abbo, K. Sposato);; Florida International University Robert Stempel College of Public Health and Social Work, Miami (A. Jimenez); U; niversity of Miami Miller School of Medicine, Miami (L. M. Abbo, O. Martinez, B. Shukla);; University of Pittsburgh School of Medicine, Pittsburgh, Pennsylvania, USA (A. Iovleva, E. L. Fowler, C. L. McElheny, Y. Doi)

**Keywords:** KPC-3, carbapenemase, *Serratia marcescens*, long-term care, beta-lactamase, carbapenem-resistant Enterobacteriaceae, infection control, bacterial proteins, disease outbreaks, bacteria, Florida, infections, antimicrobial resistance, United States, CPE, Klebsiella pneumoniae carbapenemase, KPC

## Abstract

We describe an outbreak caused by *Serratia marcescens* carrying *bla*_KPC-3_ that was sourced to a long-term care facility in Florida, USA. Whole-genome sequencing and plasmid profiling showed involvement of 3 clonal lineages of *S. marcescens* and 2 *bla*_KPC-3_-carrying plasmids. Determining the resistance mechanism is critical for timely implementation of infection control measures.

*Serratia marcescens* has been linked to healthcare-associated outbreaks, particularly after use of colistin, which is intrinsically resistant to polymyxins (*1**,**2*). Outbreaks of carbapenemase-producing *Enterobacteriaceae* (CPE) in long-term care facilities (LTCF) have been well described (*3**,**4*); outbreaks of the closely related carbapenemase-producing (CP)–*S. marcescens* are unusual. We describe an outbreak in 2 hospitals in Florida, USA, of *S. marcescens* producing *Klebsiella pneumoniae* carbapenemase (KPC). Subsequent investigation identified a local LTCF as the source.

## The Study

In June 2018, a 382-bed hospital that is part of a large hospital health system network in Miami, Florida, identified an increase of CP–*S. marcescens*. A retrospective search for more cases included all patients admitted to any facility in the 4-hospital network during October 2017–June 2018 using the automatic surveillance system (VigiLanz; VigiLanz Corporation, https://vigilanzcorp.com) with interface to the electronic medical record (EMR).

We defined cases as patients with carbapenem-resistant *S. marcescens* by Clinical and Laboratory Standards Institute (CLSI) breakpoints (*5*) isolated from any source, including clinical or surveillance cultures, during October 2017–December 2018. Based on Centers for Disease Control and Prevention guidelines, community-onset events (CO) were those cases identified <3 days after hospital admission; hospital-onset (HO) were those for which the specimens were collected >4 days after hospital admission (*6*).

In response to the outbreak, and in addition to interventions in place to prevent hospital-acquired infections (Appendix Table 1), all possible cases were prospectively identified upon admission to any of the network facilities via automatic surveillance system. Transfer forms and regular communication with the local Department of Health (DOH) notified hospitals when a known case-patient was transferred from the LTCF. All patients admitted from the source LTCF were placed in contact precautions at admission and screened for CPE. If positive, patients were placed in enhanced contact precautions (Appendix Table 1) for the duration of their stay. Miami-Dade DOH provided infection prevention and control education and support to the LTCF.

We performed matrix-assisted laser desorption/ionization time-of-flight (MALDI-TOF) mass spectrometry (bioMérieux, https://www.biomerieux-diagnostics.com) and Biofire BCID panel (bioMérieux) for bacterial identification. We conducted susceptibility testing using Vitek2 (bioMérieux) following CLSI guidelines. We tested carbapenemase production with CarbaNP test (Hardy Diagnostics, https://hardydiagnostics.com). We processed active surveillance testing (AST) using a 10 μg meropenem disk on MacConkey plate after broth enrichment; possible CPE colonies were identified and tested for carbapenemase production.

We subjected 1 isolate per patient to whole-genome sequencing on NextSeq500 (Illumina, https://www.illumina.com). We assembled sequences by SPAdes version 3.13 (https://github.com/ablab/spades) and annotated them by prokka version 1.14 (https://github.com/tseemann/prokka). Snippy version 4.4.0 (https://github.com/tseemann/snippy) was used to identify SNPs. We used ResFinder 3.2 (https://cge.cbs.dtu.dk/services/ResFinder) and PlasmidFinder 2.0 (https://cge.cbs.dtu.dk/services/PlasmidFinder) to identify antimicrobial resistance genes and plasmid replicons. We generated a SNP phylogenetic tree with RAxMLversion 8.2.11 (https://github.com/stamatak/standard-RAxML) and visualized it using Interactive Tree of Life (iTOL) version 5 (https://itol.embl.de) (*7*). We sequenced isolates 505 and 514 using the MinION platform (Oxford Science Park, UK, https://nanoporetech.com/products/minion) to define the *bla*_KPC_-harboring plasmids. We used Unicycler version 0.4.8-β (https://github.com/rrwick/Unicycler) for hybrid assembly of Illumina and MinION reads; we confirmed the presence of identified plasmids by aligning Illumina reads to the identified plasmid sequences.

We purified plasmids by alkaline-lysis method and used them to transform *Escherichia coli* TOP10 by electroporation (*8*). We selected transformants harboring *bla*_KPC-3_ on lysogenic agar with ampicillin, and confirmed acquisition of plasmids by PCR. The plasmids were extracted from the *E. coli* transformants, digested (*Eco*RI or *Hin*dIII), and run on 0.7% gel to obtain restriction patterns.

**Figure 1:**
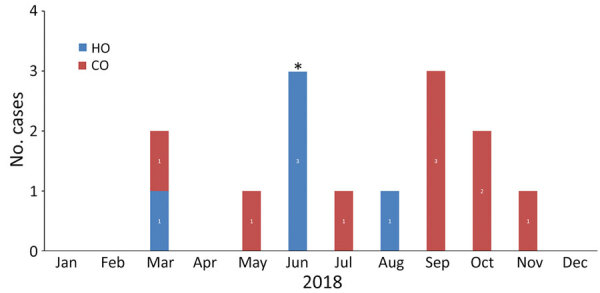
Epidemic curve of carbapenemase-producing *Serratia marcescens* infections by month in 2 hospitals of a large healthcare system in Miami, Florida, USA, 2018. Asterisk (*) indicates the implementation of new interventions in response to the outbreak. CO, community-onset; HO, hospital-onset.

During October 2017–December 2018, a total of 14 patients with CP–*S. marcescens* were identified in our hospitals (Figure 1); all patients resided at a neighboring LTCF (Table 1). Five cases (36%) were HO, but 4 were detected <15 days after admission and did not coincide in location or time with the other cases. Transmission within the hospital was not suspected; those patients were possibly colonized at admission but undetected due to low sensitivity of AST protocols. The fifth patient had long length-of-stay and previous bloodstream infection (BSI) with KPC-producing *Klebsiella pneumoniae*.

**Table 1:** Characteristics of patients with KPC-3–producing *Serratia marcescens* infection, Miami, Florida, USA*

Characteristic	**Value, N = 14**
Sex	
M	5 (35)
F	9 (65)
Median age	63 (22–89)
Median total length of stay, d	23 (3–74)
Hospital onset	5 (36)
Community onset	9 (64)
Median length of stay to positive culture, d	9 (0–73)
Hemodialysis dependent	7 (50)
Ventilator dependent (tracheostomy)	14 (100)
PEG tube	14 (100)
Positive clinical culture source	
Respiratory tract	13 (93)
Blood	3 (21)
Clinical infection	10 (71)
Pneumonia	9 (90)
Bloodstream infection	4 (40)
Colonized	4 (29)
Other MDR-GNR isolated	
Carbapenem-resistant *Pseudomonas aeruginosa*	11 (79)
KPC-producing *Klebsiella pneumoniae*	1 (7)
Carbapenem-resistant *Acinetobacter baumannii*	2 (14)
Concurrent condition	
Diabetes	5 (36)
Congestive heart failure	3 (21)
Myocardial infarction	1 (7)
ESRD	7 (50)
Dementia	1 (21)
COPD	2 (14)
CVA	8 (57)
Chronic respiratory failure	7 (50)
Hypertension	9 (64)
Discharged disposition	
Died	3 (21)
Home with home-healthcare	2 (14)
Back to source LTCF	6 (43)
Other LTCF	3 (21)
*Values are no. (%) except as indicated. COPD, chronic obstructive pulmonary disease; CVA, cerebrovascular accident; ESRD, end-stage renal disease; KPC, *Klebsiella pneumoniae* carbapenemase; LTCF, long-term care facility; MDR-GNR, multidrug resistant gram-negative rods; PEG, percutaneous endoscopic gastrostomy.	

Ten patients had >1 rectal AST; all were negative. Twelve patients had >1 tracheal aspirate AST; 2 were positive for CP–*S. marcescens* (susceptibilities in Table 2; Appendix Table 2). Ten cases had clinical infections by CP–*S. marcescens* including pneumonia (n = 9) and bloodstream infection (n = 4). Most cases were treated empirically with piperacillin/tazobactam, cefepime, and vancomycin. Targeted treatments included ceftazidime/avibactam. Four cases were colonized without signs or symptoms of CP-*S. marcescens* infection during hospital admission. Three patients died (21% in-hospital mortality); these deaths were not associated with infection by CP-*S.marcescens*.

**Table 2:** Susceptibility profiles of KPC-producing *Serratia marcescens* isolates, Miami, Florida, USA*

Drug	Total no. isolates tested	No. (%) susceptible	No. (%) intermediate	No. (%) resistant
ATM	14	0	0	14 (100)
CFZ	14	0	0	14 (100)
FEP	14	0	0	14 (100)
CAZ	14	0	0	14 (100)
CRO	14	0	0	14 (100)
LVX	14	5 (36)	1 (7)	8 (57)
MEM	14	0	0	14 (100)
AMK	14	14 (100)	0	0
GEN	14	0	13 (93)	1 (7)
TOB	14	0	0	14 (100)
SXT	14	14 (100)	0	0
TET	14	2 (14)	8 (57)	4 (28)
TGC	12	12 (100)	0	0
CZA	3	3 (100)	0	0

*AMK, amikacin; ATM, aztreonam; CAZ, ceftazidime; CFZ, cefazolin; CRO, ceftriaxone; CZA, ceftazidime/avibactam; FEP, cefepime; GEN, gentamicin; LVX, levofloxacin; MEM, meropenem; SXT, trimethoprim/sulfamethoxazole; TET, tetracycline; TGC, tigecycline; TOB, tobramycin.

During June 2018–January 2019, the 67 notifications of admissions from the source LTCF were related to 30 individual patients. In 7 cases (23%), CP–*S. marcescens* was present at admission.

**Figure 2:**
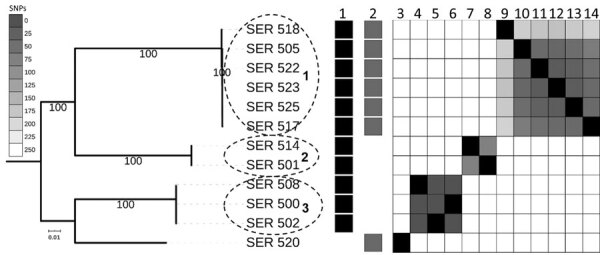
Core-genome SNP phylogeny of 12 *Serratia marcescens* isolates involved in outbreak in Miami, Florida, USA, 2018, depicted with KPC plasmid presence/absence matrix. Dotted circles indicate 3 major lineages involved in the outbreak. Nodes supported by bootstrap values of 100 are shown. A heat map of core genome SNP differences between strains involved in the outbreak shows genome similarity as measured by SNP distance; dark gray indicates higher similarity and lighter gray lower similarity. 1, pKP46-like; 2, pnJST258C2-like; 3, SER_520; 4, SER_508; 5, SER_502; 6, SER_500; 7, SER_514; 8, SER_501; 9, SER_518; 10, SER_505; 11, SER_522; 12, SER_523; 13, SER_525; 14, SER_517. Scale bar indicates number of differences between sequences. KPC, *Klebsiella pneumoniae* carbapenemase; SNP, single-nucleotide polymorphisms.

We performed molecular testing on 12 isolates, 1 per patient. Core genome analysis demonstrated the presence of 3 clonal lineages of *bla*_KPC-3_-carrying *S. marcescens* involving >1 patient, and 1 outlier (Figure 2). Eleven isolates belonging to the 3 lineages shared a 43-kb FII-type *bla*_KPC-3_-harboring plasmid, which had >99% sequence identity with pKPC_Kp46 plasmid previously described in *K. pneumoniae* (GenBank accession no. KX348146.1) (*9*); confirmation was by an identical plasmid restriction profile. Lineage 1 isolates shared a 19-kb ColRNAI-type *bla*_KPC-3_-harboring plasmid, 99% identical to previously reported pNJST258C2 from *K. pneumoniae* (GenBank accession no. CP006919.1), except for the absence of the transposable element containing aminoglycoside resistance genes *aacA1* and *aacA4* (*10*). The outlying isolate, 520, only contained a pNJST258C2-like *bla*_KPC-3_-harboring plasmid identical to that found in lineage 1. The *bla*_KPC-3_ sequence was identical between the 2 plasmids and was located on *Tn4401b*-like elements, which were identical except for a 70-bp deletion in *tnpA* leading to frameshift and premature stop in the pNJST258C2-like plasmid. This deletion did not seem to affect KPC expression; isolate 520, carrying only the pNJST258C2-like plasmid, was still resistant to carbapenems. In addition to *bla*_KPC-3_, the pKPC_Kp46–like plasmid contained *bla*_TEM-1_, *Δbla*_OXA-9_, and *qnrB19*. The pNJST258C2-like plasmid did not contain additional antimicrobial resistance genes; however, it contained an operon encoding production of colicin, an antimicrobial substance that is lethal against related strains that lack it (*11*). Isolate 520 was from the patient with a history of KPC-producing *K. pneumoniae*, suggesting that the *bla*_KPC-3_-harboring plasmid was transferred to *S. marcescens* in the patient in a separate event from the infection of the other 11 cases.

## Conclusions

Use of an automatic surveillance system enabled retrospective and prospective detection of cases and identification of their common exposure in an LTCF on the basis of shared address. Prospective identification of residents of the source LTCF enabled screening at point of entry and implementation of interventions to prevent hospital transmission. Direct communication between the infection control department and the LTCF was difficult and relied upon the local DOH to share information about known cases. Unfortunately, a regional registry of patients with CPE is not available in Florida (*12*,*13*).

The pKPC-KP4–like plasmid shared among the 3 clonal lineages involved in the outbreak, and the pNJST258C2-like plasmid, shared between lineage 1 and the outlier isolate, were the vehicles of *bla*_KPC-3_ in this polyclonal outbreak. However, it is unclear why lineage 1 isolates contained both *bla*_KPC-3_-harboring plasmids. It is possible that in addition to antimicrobial resistance, factors such as colicin production facilitated dissemination within the LTCF.

In summary, our investigation of this CP–*S. marcescens* outbreak in 2 hospitals in Florida identified a local LTCF as the source. Early identification, communication, and implementation of preventive measures within healthcare facilities and cooperation with local public health authorities are pivotal in preventing transmission of multidrug-resistant organisms among vulnerable populations.

Appendix Additional information about KPC-3–producing *Serratia marcescens* outbreak between acute- and long-term care facilities, Florida, USA.
