# Supplementary material for: KPC-3–Producing Serratia marcescens Outbreak between Acute and Long-Term Care Facilities, Florida, USA
Source: Emerg Infect Dis. 2020 Nov;26(11):2746–50. doi: 10.3201/eid2611.202203 (PMC7588513; doi:10.3201/eid2611.202203)
Supplement: Appendix — Additional information about KPC-3–producing Serratia marcescens outbreak between acute- and long-term care facilities, Florida, USA. [file 20-2203-Techapp-s1.pdf]

Article DOI: https://doi.org/10.3201/eid2611.202203

KPC-3–Producing *Serratia marcescens* Outbreak between Acute and Long-Term Care Facilities, Florida, USA

Appendix

Appendix Table 1. Interventions to prevent spread of KPC-producing *Serratia marcescens* bacteria in healthcare facilities, Florida, USA

Bundle of interventions to prevent hospital-acquired infections (HAI) in place before outbreak of *Serratia marcescens* carrying bla_{KPC3} within a large healthcare network in Miami, Florida
• Daily bathing with chlorhexidine gluconate foam, nasal decolonization with alcohol-based nasal sanitizer, and daily distribution of alcohol-based wipes for patients' hand hygiene for all adults in all units.
• AST for CPE carriage in perirectal swab and tracheal aspirate (vented patient) upon admission and weekly thereafter to all adult ICU patients.
• Enhanced contact precautions for patients infected or colonized with any CPE.
• Environmental cleaning monitoring with UV powder.
Elements of enhanced contact precautions for CPE patients
• Private room setting.
• Dedicated patient care equipment.
• Different color-coded isolation sign at the patient's room entrance.
• Patient's room cleaning with bleach-based products twice a day.
• Disposable gowns and gloves for contact isolation.
• Patient and family/visitor education.
• Staff cohorting.

AST, active surveillance testing; CPE, carbapenemase-producing *Enterobacteriales*; ICU, intensive care unit; UV, ultraviolet.

Appendix Table 2. Susceptibility testing of CZA against 3 *Serratia marcescens* carrying bla_{KPC-3} from an outbreak in Miami, Florida

Isolate	E-test MIC	Comments
ATCC 25922	0.032	
S-505	0.5	Both plasmid
S-514	0.5	KP46 plasmid only
S-520	0.047	NJST258 plasmid only

CZA, ceftazidime/avibactam. CZA MIC in isolates with 2 plasmids is not any higher than that of single plasmid, which was expected. CZA resistance usually requires KPC mutations; none were present in the isolates. The variation seen between S-520 and S-505 and S-514 was probably driven not by KPC, but by other β-lactamases, porins, or efflux. The isolates were quite distinct from each other.
